# Pax8 modulates the expression of Wnt4 that is necessary for the maintenance of the epithelial phenotype of thyroid cells

**DOI:** 10.1186/1471-2199-15-21

**Published:** 2014-09-30

**Authors:** Maria Grazia Filippone, Tina Di Palma, Valeria Lucci, Mariastella Zannini

**Affiliations:** 1 IEOS - Institute of Experimental Endocrinology and Oncology CNR - National Research Council, via S. Pansini 5, 80131 Naples, Italy

**Keywords:** Wnt4, Pax8, Transcriptional regulation, Mesenchyme-to-epithelium transition, Thyroid cancer

## Abstract

**Background:**

The transcription factor Pax8 is expressed during thyroid development and is involved in the morphogenesis of the thyroid gland and maintenance of the differentiated phenotype. In particular*,* Pax8 has been shown to regulate genes that are considered markers of thyroid differentiation. Recently, the analysis of the gene expression profile of FRTL-5 differentiated thyroid cells after the silencing of Pax8 identified Wnt4 as a novel target. Like the other members of the Wnt family, Wnt4 has been implicated in several developmental processes including regulation of cell fate and patterning during embryogenesis. To date, the only evidence on Wnt4 in thyroid concerns its down-regulation necessary for the progression of thyroid epithelial tumors.

**Results:**

Here we demonstrate that Pax8 is involved in the transcriptional modulation of Wnt4 gene expression directly binding to its 5’-flanking region, and that Wnt4 expression in FRTL-5 cells is TSH-dependent. Interestingly, we also show that in thyroid cells a reduced expression of Wnt4 correlates with the alteration of the epithelial phenotype and that the overexpression of Wnt4 in thyroid cancer cells is able to inhibit cellular migration.

**Conclusions:**

We have identified and characterized a functional Pax8 binding site in the 5’-flanking region of the Wnt4 gene and we show that Pax8 modulates the expression of Wnt4 in thyroid cells. Taken together, our results suggest that in thyroid cells Wnt4 expression correlates with the integrity of the epithelial phenotype and is reduced when this integrity is perturbed. In the end, we would like to suggest that the overexpression of Wnt4 in thyroid cancer cells is able to revert the mesenchymal phenotype.

## Background

Wnts are powerful regulators of cell proliferation and differentiation, and their signaling pathway involves proteins that directly participate in both gene transcription and cell adhesion [1]. Over the past two decades, 19 members of the Wnt protein family have been found in mammals and have been subdivided into canonical signaling, with transforming activities in mammary epithelial cells, and non canonical signaling involved in the planar cell polarity (PCP) and in Calcium signaling [2]. Member of the Wnt family, Wnt4 is classified as a non-canonical Wnt protein even though there is evidence that it is also able to activate the canonical signaling pathway [3]. Wnt4 knockdown highlighted its crucial role in the development of several organs such as kidney, ovary and mammary gland [4,5]. Moreover, Wnt4 null mice die within 24 h of birth, probably because of severe lack of kidney functions [6]. In fact, during kidney development Wnt4 plays a key role in the mesenchymal to epithelial transition and in the morphogenesis required for tubule formation [7], and its expression is regulated by the transcription factor Pax2 [8]. Interestingly, in humans defects in WNT4 are a cause of Rokitansky-Kuster-Hauser syndrome (RKH syndrome) characterized by utero-vaginal atresia in otherwise phenotypically normal female with a normal 46,XX karyotype [9]. Homozygous null mutation in WNT4 is the cause of female sex reversal with dysgenesis of kidneys, adrenals, and lungs (SERKAL syndrome), demonstrating that this gene plays an essential role in human sex-determination and organogenesis [10]. Furthermore, mutations in the WNT4 gene also cause WNT4 Müllerian aplasia and ovarian dysfunction [11].

Recently, Wnt4 was shown to be strongly down-regulated in human anaplastic carcinomas and to behave as a key factor in Ras-mediated transformation of rat epithelial cells [12]. In addition, the analysis of the gene expression profile of FRTL-5 differentiated thyroid cells after the silencing of the transcription factor Pax8 identified Wnt4 among the down-regulated genes and by ChIP assay Wnt4 was defined as a novel direct target of Pax8 [13].

Pax8, a member of the Pax genes family, was shown to be required for both morphogenesis of the thyroid gland [14] and maintenance of the thyroid differentiated phenotype [15]. Interestingly, mutations in the Pax8 gene have been associated with congenital hypothyroidism in humans [16-19]. During the embryogenesis Pax8 is expressed not only in the thyroid but also in other tissues such as the metanephros, the midhindbrain boundary region [20,21], as well as in the Müllerian duct [5]. In Pax8 knockout mice, the thyroid gland is barely visible and lacks the follicular cells; accordingly, the expression of the thyroid-specific markers thyroglobulin and thyroperoxidase cannot be detected in line with the crucial role that Pax8 plays in thyrocytes differentiation [14]. Moreover, as a consequence of their athyroidism, Pax8 deficient mice are deaf, growth retarded, ataxic, and do not survive weaning. As in most cases of congenital hypothyroid patients, in Pax8-deficient mice the symptoms can be reversed by the TH replacement therapy if instituted within the first days of postnatal life [22,23]. It is of interest to note that Pax8^-/-^ mice that were properly substituted with TH and developed otherwise normally without any overt deficits did not become fertile [24]. Indeed, the analysis of the male and female reproductive system demonstrated that the Pax8^-/-^ mice fail to develop correctly [25].

Here, we investigated the involvement of Pax8 in the regulation of Wnt4 gene expression in thyroid cells. The in silico analysis of the 5’-flanking region of the Wnt4 gene identified several putative Pax8 binding sites and our data here reported show that indeed Pax8 is involved in the transcriptional regulation of the Wnt4 gene. All together, our results indicate that in thyroid cells Pax8 participates to Wnt4 gene expression directly binding to its 5′-flanking region and that a reduced expression of Wnt4 correlates with the alteration of the epithelial phenotype. Moreover, we also show that the overexpression of Wnt4 in thyroid cancer cells is able to inhibit cellular migration.

## Results

### Wnt4 expression in thyroid cells is modulated by TSH

**Figure 1:**
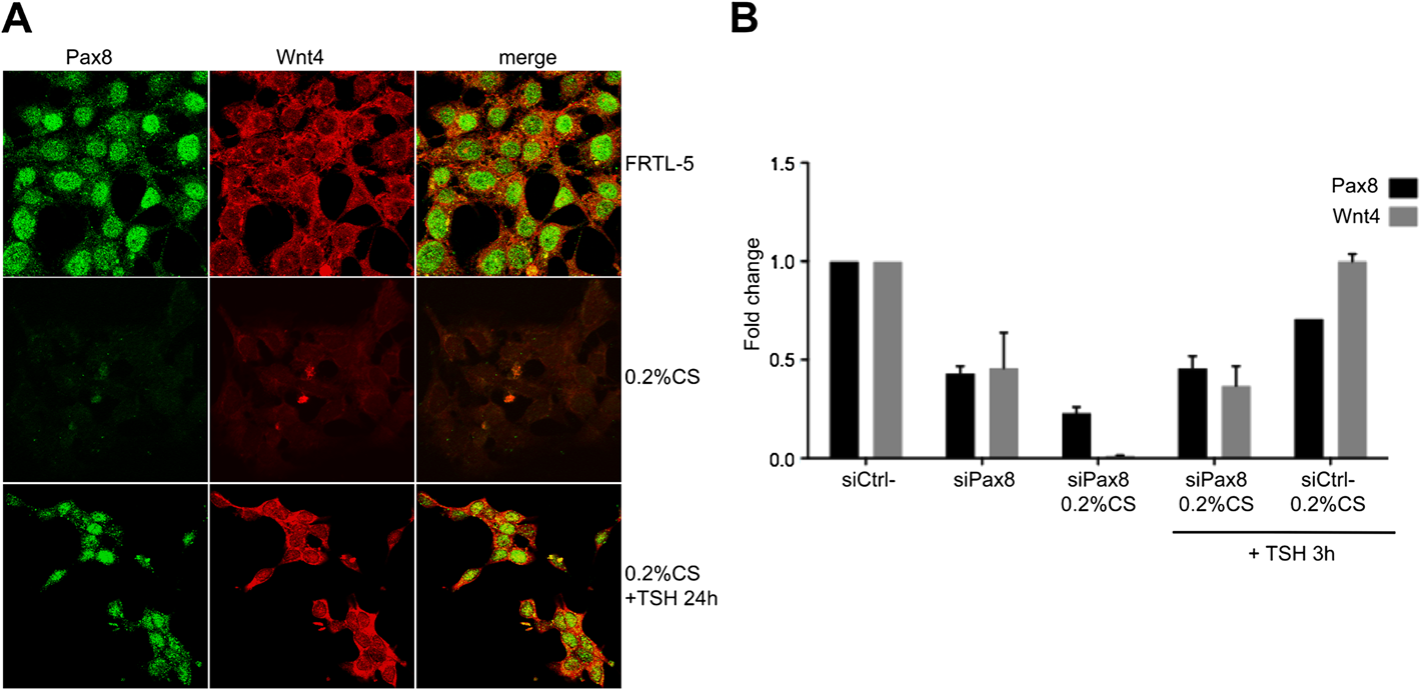
**Wnt4 is localized in the cytoplasmic compartment of FRTL-5 cells and its expression is modulated by TSH. A)** Wnt4 expression is modulated by TSH in FRTL-5 thyroid cells. FRTL-5 cells were cultured in regular medium or in starvation medium for 4 days (0.2% CS) and treated with 1 mU/ml of TSH for 24 h, and analyzed by immunofluorescence to detect Pax8 and Wnt4 proteins with specific antibodies (see Methods). **B)** TSH stimulation of Wnt4 expression is mediated by Pax8. FRTL-5 cells were cultured in regular medium and transfected with 100 nM of siPax8 or siCtrl-. Seven hours later, cells were starved in 0.2% CS medium for 24 h and treated with 1 mU/ml of TSH for 3 h and analyzed by qRT-PCR to measure the expression levels of Pax8 (black bars) and Wnt4 (gray bars) mRNAs. Data are from three independent experiments, each performed in duplicate and are expressed as the mean ± SD (P < 0,1).

In the past, we observed that in FRTL-5 cells cultured in the absence of TSH, Pax8 mRNA and protein rapidly disappear, whereas upon TSH stimulation, both mRNA and protein are newly synthesized [26]. To investigate the role of TSH in Wnt4 expression, FRTL-5 cells were starved for 4 days in 0.2% calf serum-medium and subsequently treated with TSH for 24 hours. The expression of both Pax8 and Wnt4 was analyzed at the protein level by immunofluorescence. As expected, Pax8 expression is strongly reduced upon starvation of the cells and reinduced upon TSH stimulation. Similarly, also the expression of Wnt4 turned out to be modulated by TSH in a way that well correlates with Pax8 expression profile suggesting that TSH regulation of Wnt4 expression could be mediated by Pax8 (Figure 1A).

Previous studies associated Wnt proteins with glycosaminoglycans in the extracellular matrix and bound tightly to the cell surface [27-29]. Beyond this information, the localization of Wnt proteins in vertebrates remains unclear. The immunofluorescence experiment in Figure 1A allowed us to obtain information also on the subcellular distribution of the Wnt4 protein in thyroid cells, showing the presence of Wnt4 exclusively in the cytoplasmic compartment of this cell type.To better clarify whether TSH stimulation of Wnt4 expression is entirely mediated by Pax8, we performed a Pax8 knock-down experiment. In details, FRTL-5 thyroid cells were transfected with siGENOME Pax8 siRNA (experimental) or siGENOME Non-Targeting siRNA (control), a scrambled sequence that does not show homology with any rat gene. Few hours after transfection, cells were starved in 0.2% calf serum medium for 24 hours and then stimulated for 3 hours with TSH. The expression of both Pax8 and Wnt4 was analyzed at the mRNA level by qRT-PCR. Interestingly, when Pax8 is inhibited by the silencing, the stimulation with TSH does not induce high levels of Wnt4 as it happens in the siCtrl- sample (Figure 1B). This result supports the hypothesis that a significant fraction of Wnt4 response to TSH stimulation is indeed mediated by Pax8.

### The 5′-flanking region of the Wnt4 gene is responsive to Pax8

**Figure 2:**
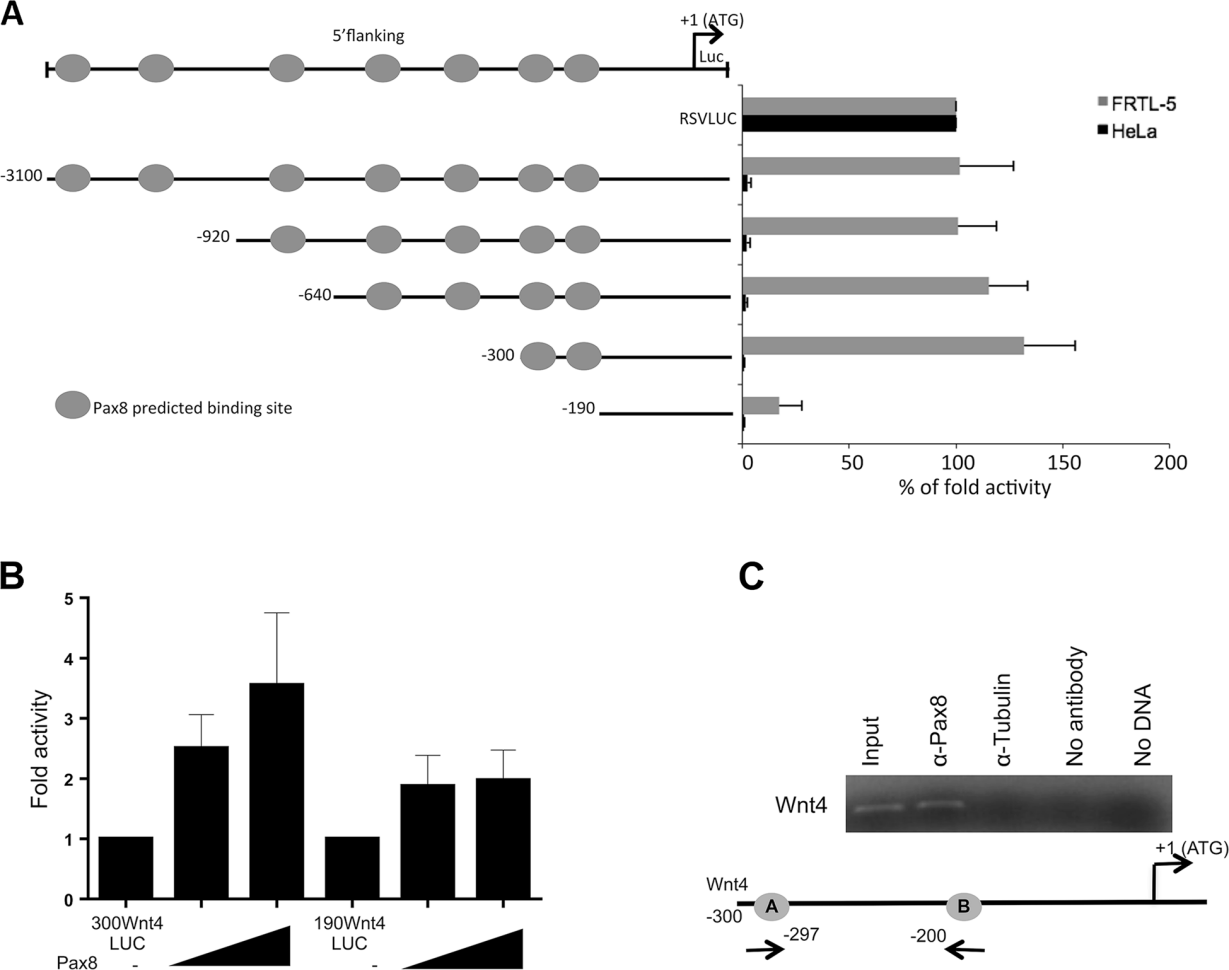
**Pax8 activates transcription from the Wnt4 5′-flanking region. A)** Transcriptional activity of Wnt4 5′-flanking genomic region. The deletion reporter constructs were transfected into FRTL-5 and HeLa cells and the luciferase activity was determined. All the constructs have the highest activity in thyroid cells and only the 190Wnt4LUC construct shows a strongly reduced activity. Data are expressed as the mean ± SD (P < 0,0001); **B)** Pax8 activates transcription from the Wnt4 promoter region. HeLa cells were transiently transfected with 300Wnt4LUC and 190Wnt4LUC reporter constructs alone and in combination with increasing concentration (100 and 200 ng) of the expression vector encoding Pax8 (CMV5-Pax8). The transcriptional activity was determined 48 h after transfection as the firefly over renilla luciferase activity. Data are expressed as fold induction over the transcription obtained with 300Wnt4LUC and 190Wnt4LUC, whose values were set at 1. Data are from three independent experiments, each performed in duplicate and are expressed as the mean ± SD (P < 0,005); **C)** Pax8 binds the Wnt4 promoter region in vivo. Chromatin extracted from cross-linked FRTL-5 cells was immunoprecipitated with an unrelated antibody or for Pax8. The immunoprecipitates were analyzed by PCR with oligonucleotides able to amplify the region between -300 bp and -190 bp of the 5′flanking region of the Wnt4 gene.

To determine whether Pax8 could regulate Wnt4 gene expression by directly binding to its regulatory genomic sequence, we performed a computational analysis using the MatInspector Software 8.0. We searched for Pax8 binding sites in a region of about 3 Kb in the Wnt4 5′-flanking region and the analysis showed the presence of several Pax8 binding sequences.To characterize more in detail the genomic region upstream the Wnt4 gene and to assess its transcriptional activity in thyroid cells, five different fragments corresponding to progressive deletions of the 5′-flanking region were subcloned in the pGL3-basic vector upstream the luciferase reporter gene. All the deletions were engineered taking into account the position of the Pax8 binding sites predicted by the MatInspector software (Figure 2A).To verify the transcriptional activity of each fragment, the deletion constructs were transiently transfected in FRTL-5 thyroid cells and in HeLa cells, the latter negative for the expression of Pax8. The results obtained demonstrate that all the constructs have a high transcriptional activity in thyroid cells. Interestingly, only the smallest construct carrying 190 bp of the Wnt4 5′-flanking region shows a strongly reduced activity in the FRTL-5 cells (Figure 2A). These data indicate that the 5′-flanking region of Wnt4 is transcriptionally active in thyroid cells and prompted us to further study the role of Pax8 in the modulation of Wnt4 expression. Initially, we tested the effect of Pax8 transient expression on the -300 and -190 promoter regions by luciferase assays (Figure 2B). Specifically, we performed transactivation assays in HeLa cells by transfecting the reporter constructs 300Wnt4LUC and 190Wnt4LUC together with increasing concentration of Pax8. As shown in Figure 2B, Pax8 is able to activate transcription from the 300Wnt4LUC reporter in a dose dependent manner but not from the smallest construct 190Wnt4LUC, suggesting that the region between -300 bp and -190 bp upstream the translational start site of the Wnt4 gene is required for Pax8 positive modulation. Subsequently, to further confirm the ability of Pax8 to physically interact with the Wnt4 promoter region in vivo, we performed chromatin immunoprecipitation (ChIP) assays on genomic DNA from FRTL-5 cells. The cross-linked chromatin was immunoprecipitated using a specific antibody against Pax8. The enrichment of the endogenous Wnt4 region was monitored by PCR amplification using specific primers designed to amplify the region of Wnt4 between -300 bp and -190 bp required for Pax8 transcriptional activation. The ChIP assay demonstrates that, in agreement with the transactivation data, Pax8 is able to bind in vivo the Wnt4 promoter (Figure 2C).

### Identification of a Pax8 binding site on the Wnt4 proximal promoter region

**Figure 3:**
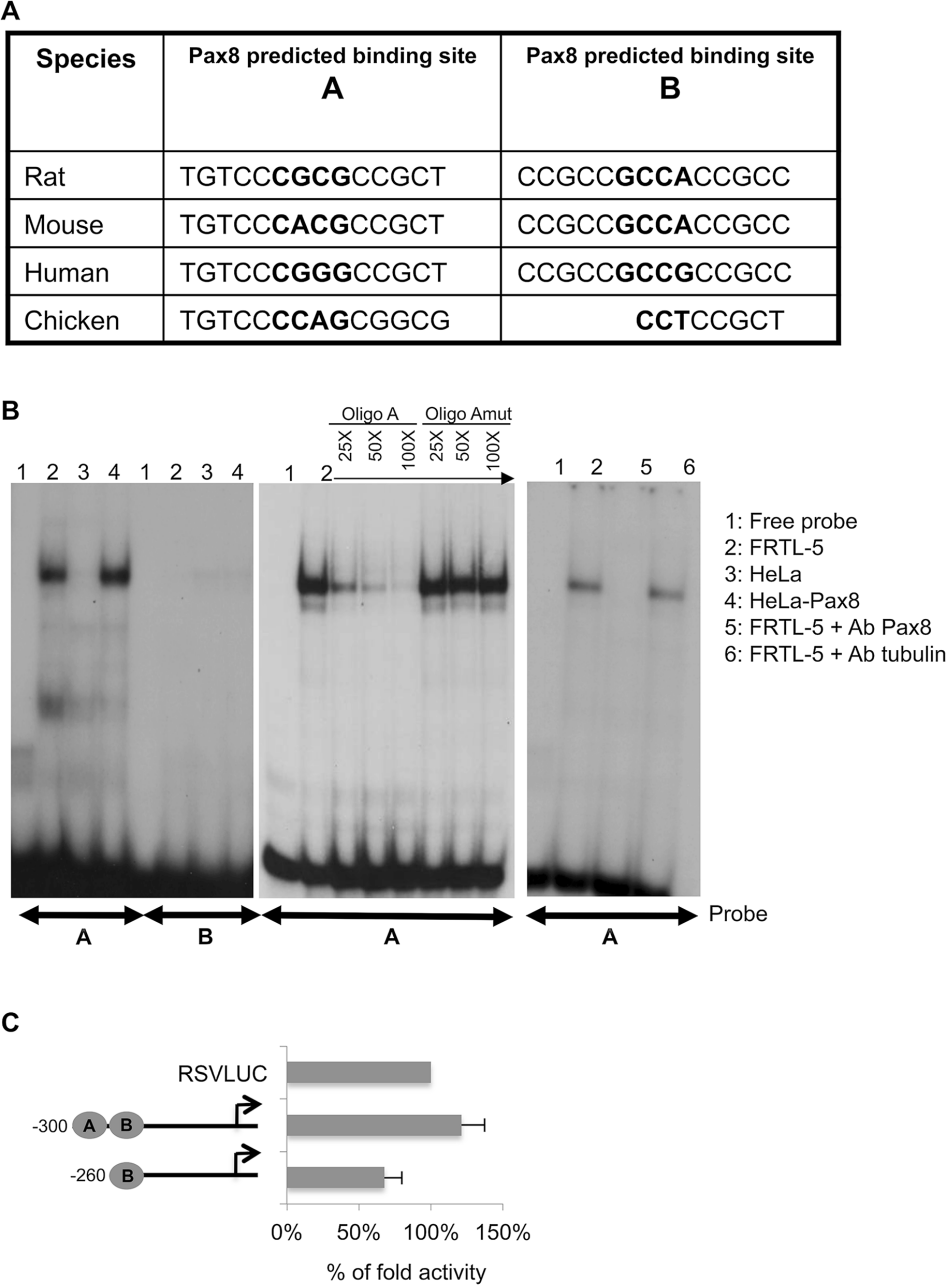
**Wnt4 5′-flanking region contains a binding site for Pax8. A)** Graphic output of the sequence analysis showing the conservation in different species of the core consensus sequences (in bold) of Pax8 binding sites. The sequence alignment was obtained using whole genome comparative analysis of the VISTA browser; **B)** Electromobility shift assays for Pax8 binding to putative recognition motifs in the Wnt4 promoter. Left panel, ^32^P-labeled oligo probes A and B were challenged with total protein extracts prepared from FRTL-5 cells (lane 2). Protein extracts of HeLa (lane 3) and Pax8-transfected HeLa cells (lane 4) were used as negative and positive control, respectively. Middle panel, the specificity of the complex observed with the FRTL-5 extract and probe A was tested by competition analysis, using increasing amount (from 25 to 100-fold molar excess) of unlabeled wild-type oligo A or mutated in the core sequence. Right panel, the FRTL-5 protein extract was incubated with the antibody against Pax8 or tubulin (as negative control), in a supershift EMSA with labeled probe A; **C)** Pax8 binding site A is necessary for the transcriptional activity of Wnt4 promoter. The deletion constructs 300Wnt4LUC and 260Wnt4LUC were transfected into FRTL-5 thyroid cells and the luciferase activity was determined. Data are from three independent experiments, each performed in duplicate and are expressed as the mean ± SD (P < 0,05).

The in silico analysis by the MatInspector software predicted two binding sites for Pax8 within the region spanning from -300 bp to -190 bp upstream the ATG of the Wnt4 gene that we named A and B, respectively. Specifically, site A was identified on the negative strand, while site B was identified on the positive strand of the DNA sequence, positioned on the rat promoter at -297 and -211 bp from the ATG, respectively. The alignment of the core sequence of the two putative Pax8 binding sites shows a high grade of conservation among the species, highlighting the potential relevance of these sites for Wnt4 expression (Figure 3A).To further characterize and verify the prediction obtained by the MatInspector analysis, we designed two oligo probes, containing the putative binding sites A and B, and we performed gel mobility shift experiments. Interestingly, the retarded band observed when the FRTL-5 protein extract was incubated with oligo A (Figure 3B, probe A, lane 2) is similar to that observed in the positive control (Figure 3B, probe A, lane 4). In contrast, the predicted binding site B could not be confirmed as a Pax8 binding site (Figure 3B, probe B). Afterwards, we demonstrated by competition and supershift assays that the shift observed when the FRTL-5 extract is incubated with oligo A is the result of a specific interaction (Figure 3B). All together, these results demonstrate that Pax8 is able to specifically bind to the sequence identified in this study.To demonstrate the functional relevance of Pax8 binding to the Wnt4 promoter, we generated a deletion mutant (260Wnt4LUC) that lacks the sequence predicted to be recognized by Pax8. Luciferase reporter constructs 300Wnt4LUC and 260Wnt4LUC were transiently transfected into FRTL-5 cells and the luciferase activity was measured 48 h later. As shown in Figure 3C, transfection of the 300Wnt4LUC construct produces high levels of luciferase activity, while the selective deletion of Pax8 binding site results in a reduced activity of the 260Wnt4LUC construct (about 50% reduction), indicating that the binding site A that we have identified is necessary for Wnt4 full promoter activity.

### Wnt4 is responsible for the maintenance of the epithelial phenotype

**Figure 4:**
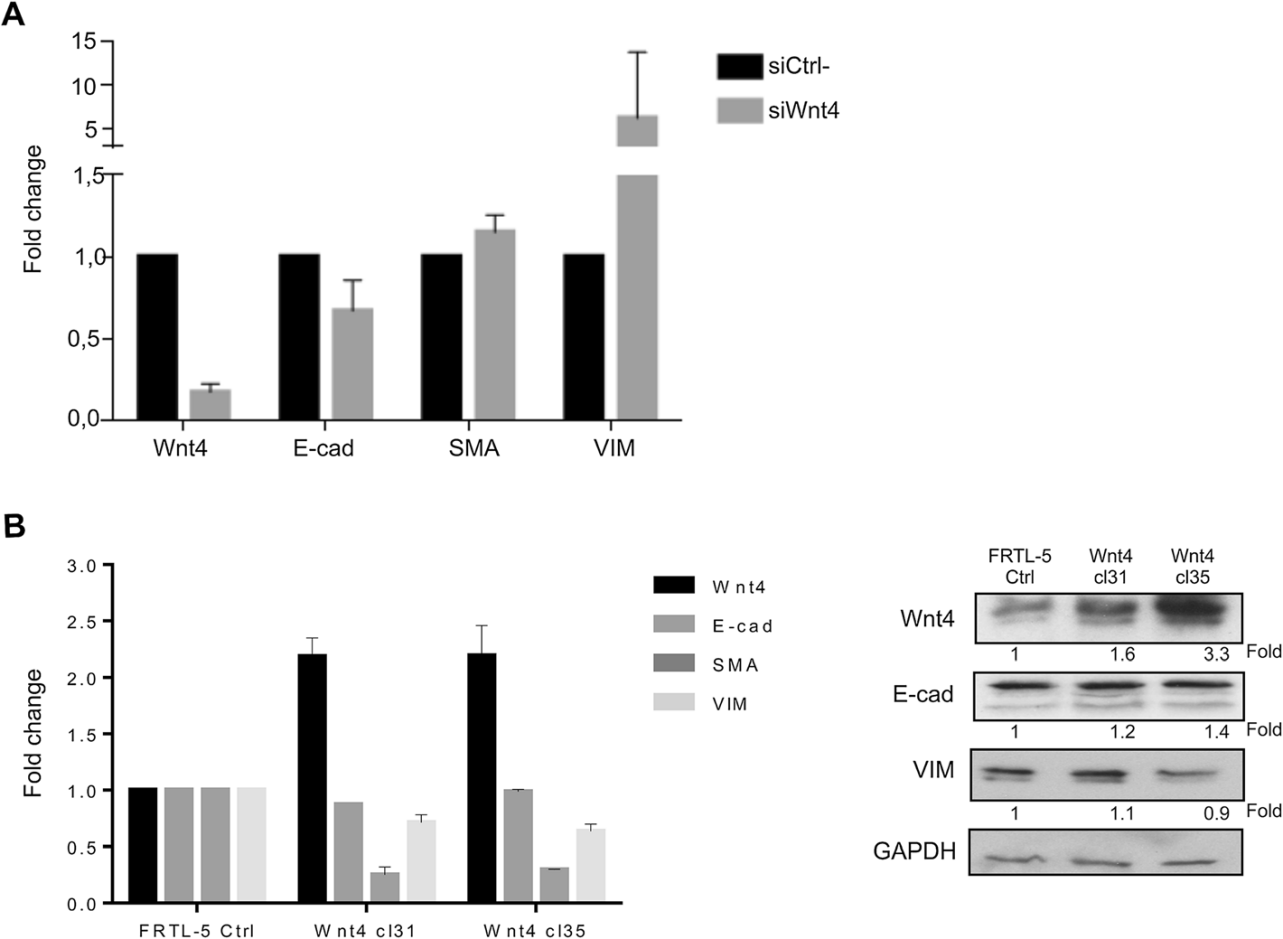
**Wnt4 expression correlates with the maintenance of the epithelial state. A)** Effect of Wnt4 silencing on EMT of thyroid cells. qRT-PCR analysis was performed on total RNA prepared from FRTL-5 cells transfected with siWnt4 or siCtrl-. The expression of E-cad, α-SMA and VIM was measured; **B)** Effect of Wnt4 overexpression on EMT of thyroid cells. qRT-PCR analysis was performed on total RNA prepared from FRTL-5-Wnt4 clones 31 and 35 and from FRTL-5 stable transfected with the empty vector. The expression of E-cad, α-SMA and Vim was measured. Data are expressed as the mean ± SD (P < 0,05). On the right, protein levels were evaluated by immunoblotting analysis using Wnt4, E-cad and VIM antibodies. The hybridization with GAPDH assessed the protein uniform loading integrity. Fold represents the quantification of protein levels normalized with GAPDH.

Recently, Wnt4 was demonstrated to be essential for normal conversion of metanephric mesenchyme to the epithelia of the nephron [6]. This evidence prompted us to investigate the involvement of Wnt4 in the EMT process also in epithelial thyroid cells. To this end, we inhibited Wnt4 expression in FRTL-5 by means of RNA interference and we analyzed by qRT-PCR the expression level of some EMT markers. Seventy-two hours after siRNA transfection, we analyzed the expression level of Wnt4, E-cadherin (E-cad) as an epithelial marker and α-Smooth muscle actin (α-SMA) and Vimentin (VIM) as mesenchymal markers. By qRT-PCR we show that upon Wnt4 knockdown, the mRNA level of E-cad is reduced while the expression level of the mesenchymal markers α-SMA and VIM is upregulated with respect to the FRTL-5 transfected with siCtrl- (Figure 4A).Our findings demonstrate that reduced levels of Wnt4 correlate with EMT, suggesting a relevant role of this gene in the maintenance of the epithelial phenotype of FRTL-5 thyroid cells. To better clarify the involvement of Wnt4 in this process, we generated FRTL-5 cells overexpressing Wnt4 and we analyzed, by qRT-PCR and Western blot, the expression level of the EMT markers above mentioned. The stable overexpression of Wnt4 is able to revert the mesenchymal phenotype; in fact, the upregulation of Wnt4 determines a stabilization of E-cad and a reduction of α-SMA and VIM (Figure 4B). The apparent difference between mRNA and protein level of VIM in our opinion could be due to the biological differences between transcript and protein abundance, underlining the importance of post-translational mechanisms controlling gene expression.

### Wnt4 is down-regulated in human thyroid cancer cells and its overexpression inhibits cellular migration

**Figure 5:**
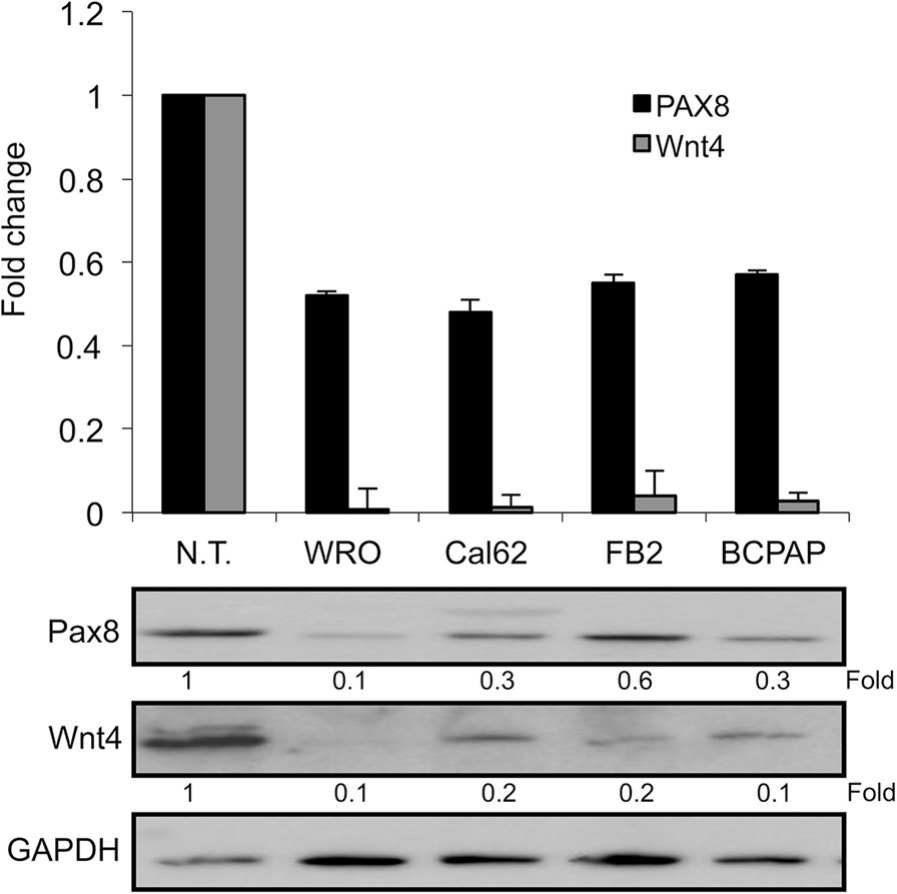
**Pax8 and Wnt4 expression in human thyroid cancer cell lines.** Wnt4 and Pax8 expression was measured by qRT-PCR in four human thyroid cancer cell lines: WRO from follicular thyroid cancer, Cal62 from anaplastic thyroid carcinoma, FB2 and BCPAP from papillary thyroid carcinoma. RNA from six non-pathological thyroids (N.T.) was used as control. Quantitative real-time PCR was carried out in triplicate as described in Materials and Methods. Glyceraldehyde 3-phosphate dehydrogenase was used as reference gene; results are reported as 2^-∆Ct^. Data are expressed as the mean ± SD (P < 0,05). The bottom panel shows the total protein extracts of FRTL-5 cells (used as normal thyroid) and human thyroid cancer cells WRO, Cal62, FB2 and BCPAP separated on SDS-PAGE and subjected to Western blot analysis with specific antibodies as indicated in the panel. The hybridization with GAPDH assessed the protein uniform loading integrity. Fold represents the quantification of protein levels normalized with GAPDH.

**Figure 6:**
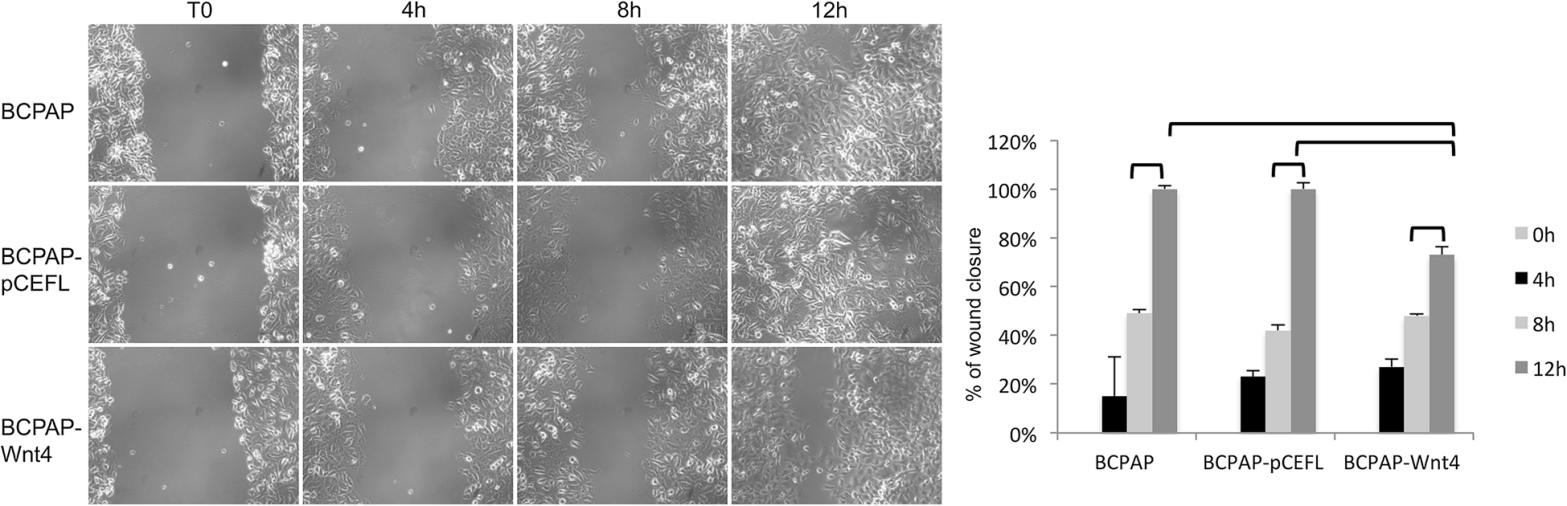
**Wnt4 overexpression in human papillary thyroid cancer cells inhibits cellular migration.** Wound healing migration assay for BCPAP, BCPAP-pCEFL and BCPAP-Wnt4 was performed. The healing of the wounds by migrating cells was imaged at time 0 h, 4 h, 8 h and 12 h (upper panel). BCPAP-Wnt4 cells migrate significantly less than BCPAP and BCPAP-pCEFL cells. Wound closure is expressed as the percentage of the initial wound diameter at 0 h. Data are expressed as the mean ± SD (P < 0,01, right panel).

We analyzed the expression level of Wnt4 in human thyroid cancer cell lines by qRT-PCR. In particular, we analyzed the WRO, Cal62, FB2 and BCPAP cells derived from follicular, anaplastic and papillary thyroid carcinoma, and a pool of six normal thyroid (N.T.) tissues as control. As expected, in all the thyroid cancer cell lines the expression level of Wnt4 is strongly reduced with respect to normal thyroid (Figure 5), indicating that a down-regulation of Wnt4 occurs in thyroid carcinoma. Interestingly, also the expression of PAX8 is significantly down-regulated in the same cell lines. The same result has been obtained by Western blot analysis (Figure 5, bottom panel). To further evaluate the involvement of Wnt4 in thyroid tumors we overexpressed Wnt4 in the BCPAP cell line and we selected a BCPAP-Wnt4 mass population. In parallel, we also generated a control mass population transfected with the backbone vector (BCPAP-pCEFL).To study whether elevated levels of Wnt4 might have a role in cell migration, we performed wound-healing assays using the two mass populations described above. The assay shows that after 12 hours the area of the wound is completely recovered by the BCPAP parental and the BCPAP-pCEFL cells. At difference, the motility of the BCPAP-Wnt4 cells appears strongly impaired, suggesting that Wnt4 overexpression significantly reduces the migration ability of human papillary thyroid cells (Figure 6).

## Discussion

In the last few years many data revealed that a set of transcriptional regulators, unique to the thyroid follicular cell type, has been identified as responsible for thyroid-specific gene expression. It comprises three transcription factors: TTF-1, Foxe-1 and Pax8, each of which is expressed also in cell types different from the thyroid follicular cells. Albeit, the specific combination of these factors is unique to the thyroid hormone producing cells, strongly suggesting that such combination plays an important role in the differentiation process of these cells [30,31]. In particular, the transcription factor Pax8 is a master gene for thyroid differentiation, being necessary for the transcriptional activation of all the known differentiation markers [32]. Moreover, Pax8 knockout mice show a severe thyroid phenotype [14] and are unfertile [24,25]. In humans, patients carrying mutations in the Pax8 gene suffer from congenital hypothyroidism [16,33].

Together with the transcription factor Pax2, Pax8 is also a central regulator of both nephron differentiation and branching morphogenesis in the developing kidney. Both transcription factors are necessary and sufficient for the formation of the pronephros and all subsequent kidney structures, regulating key target genes involved in pro/mesonephros formation [20,34]. In parallel, a cooperative role for Pax2 and Pax8 in metanephric branching morphogenesis and nephron differentiation has been uncovered [34].

In thyroid, Wnt4 has been recently demonstrated to be a key player in Ras-mediated transformation of epithelial cells. Moreover, our recent data suggested that Wnt4 could be a direct target of Pax8 in FRTL-5 rat thyroid cells [13].

In this study, we show that Pax8 modulates the expression of Wnt4 at the transcriptional level. By EMSA, ChIP and transfection assays, we demonstrate that Pax8 directly binds to the Wnt4 promoter region and activates transcription from it.

It is well known that TSH is the main regulator of thyroid differentiation [26]. Its role in this process has been studied in different model systems [35] and the expression of several thyroid-specific genes, such as Tg, TPO, TSHR and NIS has been shown to be modulated by TSH/cAMP. In the past, we demonstrated that in PCCl3 cells, TSH regulates the expression of Tg and Pax8 at the transcriptional level by a cAMP-mediated mechanism [26]. Here, we show that in FRTL-5 rat thyroid cells also the expression of Wnt4 is under the control of TSH and that this control is likely mediated by Pax8. In fact, TSH deprivation causes a significant reduction of both Pax8 and Wnt4 and the kinetic of induction of Wnt4 expression upon TSH stimulation well correlates with that of Pax8. Interestingly, it has been demonstrated that TSH supports maintenance of thyroid follicular integrity in primary cultured pig thyrocytes, stabilizing E-cadherin at the cell surface and preventing its accelerated turnover to support the maintenance of thyroid follicular integrity [36]. Therefore, we would like to propose that TSH promotes and ensures the maintenance of the architecture and integrity of the follicles also regulating the expression of Wnt4. Of course, this is currently a hypothesis that requires further investigations and experimental validations.

The epithelial-mesenchymal transition (EMT) is an orchestrated series of events in which cell-cell and cell-extracellular matrix (ECM) interactions are altered to release epithelial cells from the surrounding tissue. The cytoskeleton is reorganized to confer the ability to move through a three-dimensional ECM, and a new transcriptional program is induced to maintain the mesenchymal phenotype. Induction of EMT can compromise the mechanical and physiological integrity of the tissue, and inappropriate induction of this process can have disastrous consequences. EMT also acts in tumor progression by providing increased resistance to apoptotic agents [37,38] and by producing supporting tissues that enhance the malignancy of the central tumor.

Recently, Wnt4 has been demonstrated to be critical for mesenchyme – epithelium transition of the epithelia of the nephron [6]. In agreement with these data, we report in this manuscript that in FRTL-5 thyroid cells Wnt4 is involved in the maintenance of the epithelial phenotype. In fact, the EMT induced by Wnt4 down-regulation can be reverted in the cellular model system that stable overexpresses Wnt4. These results allow us to suggest that in thyroid cells, as in kidney cells, the expression of Wnt4 correlates with the integrity of the epithelial phenotype.

Many publications emphasize the role of the Wnt/β-catenin pathway in thyroid cancer. Recently, it has been shown that Wnt4 down-regulation is necessary for the progression of thyroid epithelial tumors toward a fully malignant phenotype [12]. Moreover, it has been extensively demonstrated that a progressive decrease of Pax8 level occurs in thyroid tumors from follicular adenoma to differentiated carcinoma and then to anaplastic carcinoma, which parallels the progressive dedifferentiation and increasing malignancy of thyroid tumors [39]. Here, we show that in human thyroid cancer cell lines, Wnt4 and PAX8 mRNA levels are strongly reduced. Moreover, the overexpression of Wnt4 in human papillary cancer cells is able to affect the stabilization of the epithelial state and to inhibit cellular migration.

In the future, it will be interesting to further clarify the role of Wnt4 in thyroid differentiation and tumorigenesis.

## Conclusions

The results presented in this study demonstrate that the transcriptional factor Pax8 is involved in the transcriptional modulation of Wnt4 gene expression in differentiated thyroid cells. Furthermore, we show that a reduced expression of Wnt4 correlates with the alteration of the epithelial phenotype and that Wnt4 overexpression in thyroid cancer cells is able to inhibit cellular migration. Taken together, our data highlight a potential intriguing role of Wnt4 in the context of the thyroid cell.

## Methods

### Protein extracts and Western blot

Cells were washed twice with ice cold PBS and lysed in a buffer containing 50 mM HEPES (pH 7.5), 150 mM NaCl, 5 mM EGTA (pH 7.8), 10% glycerol, 1% triton X-100, 1 mM DTT and 1 mM PMSF. Protein concentration was determined using the Bio-Rad protein assay (Bio-Rad Laboratories, Inc., Hercules, CA). For Western Blot analysis, proteins were separated by SDS-PAGE. Gels were blotted onto Immobilon P (Millipore Corp., Bedford, MA) for 3 h, and the membranes were blocked in 5% nonfat dry milk for 1 h at room temperature. The antibodies used for immunoblotting were: Wnt4 (Mouse anti-WNT4, Invitrogen), Pax8 (kindly provided by Prof. R. Di Lauro), vimentin, E-cadherin, GAPDH (Santa Cruz Biotechnology, Inc.). Subsequently, the filters were developed using an enhanced chemi-luminescence detection method (Pierce Chemical Co., Madison, WI) according to the manufacturer’s directions.

### Indirect immunofluorescence

FRTL-5 cells, grown on 12 mm diameter glass coverslips, were fixed with pre-chilled methanol at -20°C for 5 min, permeabilized for 10 min in 0.2% triton X-100 in PBS, and then rinsed with phosphate-buffered saline (PBS) to prepare them for immunostaining. Briefly, the cells were subsequently blocked with washing buffer containing 0.5% BSA for 1 h and treated with rabbit policlonal anti-WNT4 (Wnt4 antibody, Novus) O/N and then with Alexa Fluor^®^ 594-conjugated secondary antibody (Invitrogen) for 30 min, followed by treatment with mouse monoclonal anti-PAX8 (BIOCARE MEDICAL) for 1 h and finally by an Alexa Fluor^®^ 488-conjugated secondary antibody (Invitrogen). Several washes were interposed between the different antibodies incubations. Cell nuclei were identified by Hoechst staining. The coverslips were mounted on a microscope slide using a 70% (v/v) solution of glycerol in PBS and images were collected as previously described [40].

### Promoter constructs and plasmids

Specific proximal promoter fragments of rat Wnt4 gene were amplified by PCR. The 3100Wnt4LUC was subcloned into MluI/BglII sites of the pGL3basic vector (Promega, Madison, WI), while all the other constructs were subcloned into XhoI/HindIII of the same vector.

Wnt4 cDNA (NCBI Reference Sequence NM_005923) was kindly provided by G. De Vita. The CMV-Pax8 construct was already described [41].

### Cell culture and transfection assays

Rat thyroid follicular FRTL-5 cells were maintained in Coon’s modified F12 medium (EuroClone, MI, Italy) as previously described [42].

The starvation medium consisted of Coon’s modified Ham’s F12 medium supplemented only with 0.2% newborn calf serum (HyClone, Logan, UT). TSH treatment was performed by the addition of 1 mU/ml TSH (Sigma-Aldrich, St. Louis, MO) to the culture medium at 3 h or 24 h after starvation.

HeLa, WRO, FB2, Cal62 and BCPAP cells were grown in DMEM (EuroClone) supplemented with 10% (v/v) fetal bovine serum (HyClone, Logan, UT).

Transfections and luciferase assays were carried out as previously described [43].

### ChIP assay

ChIP was performed as previously described [13].

Precleared chromatin from FRTL-5 cells was incubated with 1 μg of affinity-purified rabbit polyclonal antibody anti-Pax8 (kindly provided by Prof. R. Di Lauro), unrelated antibody (α Tubulin, Santa Cruz) or no antibody and rotated at 4°C for 16 h. The immunoprecipitated DNA was analyzed by PCR using the following Wnt4 primers: 5′- GATCCAGAAGCGAGGTTTCGGAT -3′ and 5′-AACCTAGTCACTAGCGCTCGGG-3′.

### Electrophoretic mobility shift assay (EMSA)

Double-strand oligonucleotides were labeled with γ-^32^P ATP and T4 polynucleotide kinase and used as probes. The binding reactions were carried out in a buffer containing 20 mM Tris–HCl (pH 7.6), 75 mM KCl, 1 mM dithiothreitol, 10% glycerol, 1 mg/ml BSA, and 3 mg/ml polydeoxyinosinic deoxycytidylic acid. After 30 min of incubation at room temperature, free DNA and DNA-protein complexes were resolved on a 6% non-denaturating polyacrylamide gel and visualized by autoradiography.

The oligonucleotides used in the competition assay and the antibodies used in the supershift experiments were incubated with the protein extract for 20 min before adding the probe.

Oligonucleotides were: probe A, 5′-GCGGGTCCAGCGGCGCGGGACACCCCCC-3′ and probe B, 5′-TCCGCCGCCACCGCCGCATCCCGGCTCTG-3′. The oligonucleotide mutated in the core sequence was: 5′-GCGGGTCCAGCGGATATGGACACCCCCCC-3′.

### RNA interference

FRTL-5 cells were plated (8×10^4^ cells) in a 24-well plate and were transfected in triplicate with 50 nM rat Wnt4 siRNA (Ambion, Life Technologies Ltd, UK), 50 nM siGENOME Non-Targeting #3 (Dharmacon, Lafayette, CO), or with 100 nM Pax8 siGENOME siRNA or siGENOME Non-Targeting #3 (DHARMACON) as scramble, using the DharmaFECT1 transfection reagent, following the manufacturer’s protocol.

### RNA extraction, cDNA synthesis and quantitative RT-PCR

Total RNA was prepared using TRIzol Reagent (Invitrogen, San Diego, CA) according to the manufacturer’s directions. Total RNA (1 μg) was retrotranscribed using the iScript cDNA Synthesis kit (Bio-Rad Laboratories). Real-time PCR analysis was performed using an iCycler-iQ real-time detection system and SYBR green chemistry (Bio-Rad Laboratories).

Reactions were carried out in duplicate in three independent experiments. For each gene, values are means ± SD of three independent experiments, normalized by the expression of an housekeeping gene, and expressed as a percentage of the value measured in parental FRTL-5 or BCPAP cells. To calculate the relative expression levels, we used the 2^-^DDCT method [44].

### Wound-healing assay

Confluent BCPAP, BCPAP-pCEFL and BCPAP-Wnt4 cells plated on tissue culture dishes were wounded by manual scratching with 10-μl pipette tip, washed with PBS and incubated at 37°C in complete media. At the indicated time points, phase contrast images at specific wound size were captured.

## Abbreviations

Pax8: Paired box transcription factor 8; Wnt4: Wingless-type MMTV integration site family, member 4; EMSA: Electro mobility shift assay; UTR: Untranslated region; CS: Calf serum; EMT: Epithelial to mesenchymal transition; TSH: Thyroid stimulating hormone; ChIP: Chromatin immuno precipitation; PCP: Planar cell polarity; Tg: Thyroglobulin; TPO: Thyroperoxidase; E-cad: E-cadherin; SMA: α-smooth muscle actin; VIM: Vimentin; NIS: Sodium-iodide symporter; ECM: Extracellular matrix.

## Competing interests

The authors declare that they have no competing interests.

## Authors’ contributions

MGF carried the majority of the experiments and drafted the manuscript, TDP participated in the design of the study and carried out the ChIP assays, VL carried out the immunofluorescence analysis, MZ was responsible for the coordination and supervision of the entire study. All authors read and approved the final manuscript.
